# The impact of enoxaparin on ovarian torsion–detorsion damage in rats

**DOI:** 10.1590/acb414526

**Published:** 2026-08-03

**Authors:** Eren Altun, Duygu Lafci, Ceyda Sancakli Usta, Mustafa Hilmi Yaranoglu, Eray Metin Guler

**Affiliations:** 1 University of Health Sciences – Bağcılar Training and Research Hospital – Department of Pathology – Istanbul – Türkiye.; 2 Balikesir University – School of Medicine – Department of Obstetrics and Gynecology – Balikesir – Türkiye.; 3 Beylikduzu State Hospital – Clinic of Obstetrics and Gynecology – Istanbul – Türkiye.; 4 Balikesir University – Experimental Research Center – Balikesir – Türkiye.; 5 Haydarpasa Numune Health Application and Research Center – Clinic of Medical Biochemistry – Istanbul – Türkiye.; 6 University of Health Sciences – Faculty of Hamidiye Medicine – Department of Medical Biochemistry – Istanbul – Türkiye.

**Keywords:** Enoxaparin, Ischemia, Ovarian Torsion, Oxidative Stress, Reperfusion Injury

## Abstract

**Purpose:**

This study aimed to evaluate the protective effects of enoxaparin on ovarian tissue subjected to experimental ischemia/reperfusion (I/R) injury in rats.

**Methods:**

Thirty female rats were divided into three groups: control, ischemia/reperfusion (I/R), and ischemia/reperfusion plus enoxaparin (I/R+E). Ovarian I/R injury was induced by 1 hour of ischemia followed by 1 hour of reperfusion. Rats in the I/R+E group received subcutaneous enoxaparin (0.5 mg/kg) prior to ischemia. Histopathological injury was assessed using a standardized scoring system, and total antioxidant status (TAS), total oxidant status (TOS), and oxidative stress index (OSI) were measured.

**Results:**

Histopathological injury scores were significantly higher in the I/R group compared with controls (*p* < 0.05), with no significant difference between the I/R and I/R+E groups (*p* > 0.05). Biochemical analysis showed significantly higher TAS and lower OSI levels in the I/R+E group compared with the I/R group (*p* < 0.05), while TOS levels were comparable between groups.

**Conclusion:**

Although enoxaparin did not significantly improve histopathological injury, its beneficial effects on oxidative stress parameters suggest a protective role against oxidative stress in ovarian I/R injury.

## Introduction

Ovarian torsion is a frequent gynecological emergency in women of reproductive age, requiring prompt surgical intervention to preserve ovarian function. However, accumulating evidence suggests that detorsion itself may exacerbate ovarian tissue injury through ischemia–reperfusion (I/R) mechanisms^1,2^. Reduced blood flow during torsion leads to tissue hypoxia, while subsequent reperfusion results in excessive production of reactive oxygen species (ROS), causing lipid peroxidation, mitochondrial dysfunction, nuclear damage, and ultimately follicular loss via apoptosis or necrosis^3^.

These findings indicate that surgical detorsion alone may be insufficient to prevent ovarian damage and loss of ovarian reserve. Consequently, adjunct pharmacological strategies targeting oxidative stress and inflammation have gained increasing attention. Several experimental studies have demonstrated the potential protective effects of anti-inflammatory and antioxidant agents in ovarian I/R injury models^4,5^. Notably, it has been reported that detorsion without additional medical therapy fails to adequately preserve ovarian reserve, highlighting the need for effective cytoprotective interventions^6^.

Enoxaparin sodium, a low-molecular-weight heparin widely used in obstetrics and gynecology, has been shown to exert protective effects on ovarian reserve in experimental ovarian torsion–detorsion models^6^. Beyond its anticoagulant properties, enoxaparin is also reported to possess antioxidant and anti-inflammatory effects. Nevertheless, its impact on systemic oxidative balance, particularly total oxidant status (TOS) and total antioxidant status (TAS) following ovarian I/R injury, remains poorly defined. Moreover, the optimal strategy for preserving ovarian tissue and the follicular pool following I/R injury remains under debate. Although previous studies have shown no significant effect of enoxaparin on specific antioxidant enzymes, such as glutathione and superoxide dismutase, a comprehensive evaluation of its global oxidative effects is lacking^7^.

Therefore, the present study aimed to evaluate the histopathological ovarian damage and systemic oxidative stress response, assessed by plasma TOS and TAS levels, following enoxaparin administration in a rat model of ovarian I/R injury.

## Methods

This study was approved by the Balikesir University Animal Experiments Local Ethics Committee (approval number 2024/10-4). The ethical approval explicitly included permission to collect ovarian tissue and blood samples (serum and plasma). Following completion of the approved protocol, tissue use authorization was confirmed, and all experimental procedures were conducted in accordance with the Directive of the Balikesir University Animal Experiments Local Ethics Committee.

A total of 30 female Wistar albino rats weighing 210–250 g were included in the study. Sample size calculation was performed using power analysis (alpha error = 0.05; 1–beta = 0.8), and a minimum of nine rats per group was required. An experimental ovarian I/R model, previously described and validated in the literature, was employed^8,9^. The rats were randomly assigned to three groups, each consisting of 10 animals:

- Group 1: Control group (n = 10);
- Group 2: I/R group (n = 10);
- Group 3: I/R plus enoxaparin group (I/R+E; n = 10).

All animals were housed under controlled environmental conditions with a constant temperature (22 ± 1°C) and a 12 h light/12 h dark cycle, with free access to standard laboratory chow and water. Only rats exhibiting at least three regular estrous cycles were included in the study. Estrous cycle determination was performed using the vaginal smear method as described by Marcondes et al.^10^. To ensure homogeneity among groups, all animals were included during the diestrus phase.

All rats were anesthetized with ketamine hydrochloride (80 mg/kg) and xylazine hydrochloride (10 mg/kg) administered intramuscularly. Anesthesia was maintained with additional doses of ketamine (50 mg/kg) and xylazine (10 mg/kg) as required. After induction, animals were placed in the supine position, the abdominal area was shaved, and skin disinfection was performed using 10% povidone–iodine.

**Figure 1:**
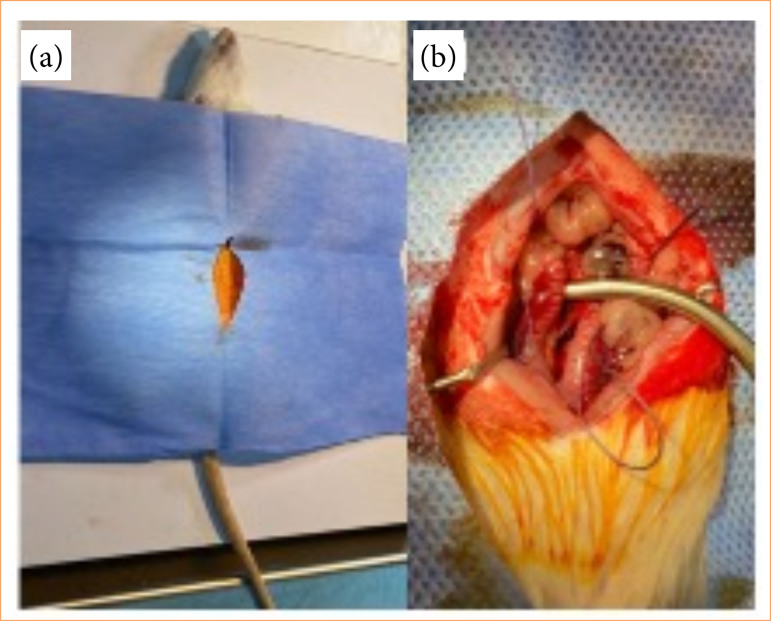
Surgical procedure. (a) Surgical preparation. (b) Clamping of the distal abdominal aorta using a Bulldog clamp to induce ischemia and bilateral ovarian ligation performed with Vicryl sutures.

Source: Elaborated by the authors.

A 2–3 cm lower midline abdominal incision was made to perform laparotomy (Fig. 1). To prevent collateral circulation, bilateral ovaries were ligated with polyglycolic acid sutures using 3-0 to 4-0 Vicryl sutures (Vicryl; Johnson & Johnson Medical, Ethicon). During the 1-hour ischemic period, the incision site was covered with moistened gauze to minimize heat and fluid loss. After the designated ischemic period, the sutures were carefully removed to restore collateral circulation, and ovarian reperfusion was achieved.

In the I/R groups, ischemia was maintained for 1 hour, followed by 1 hour of reperfusion. In the I/R + enoxaparin group, ischemia was induced using the same protocol, but subcutaneous enoxaparin (0.5 mg/kg) (Clexane 4,000 anti-Xa/0.4 mL, Sanofi) was administered 2 hours prior to ischemia induction. This dose was selected based on previous studies demonstrating equivalence to the 40-mg/day prophylactic dose used in humans^11^.

In the I/R and I/R + enoxaparin groups, ovaries were excised immediately after reperfusion. Animals were euthanized intraoperatively by exsanguination, and blood samples were collected for serum analysis.

### Histologic evaluation

Paraffin-embedded ovarian tissues were sectioned at a thickness of 3–4 μm using a microtome (Leica RM2135, Wetzlar, Germany). The sections were subsequently stained with Harris hematoxylin (Merck, Darmstadt, Germany) and Eosin G (Merck, Darmstadt, Germany) for routine hematoxylin and eosin (H&E) evaluation.

**Figure 2:**
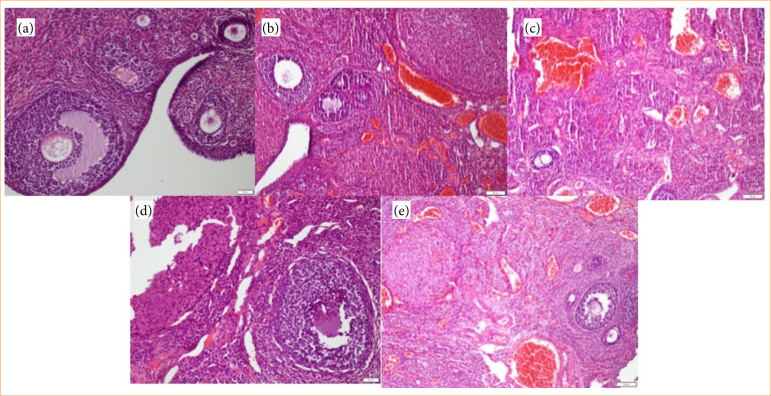
Histopathological morphological changes. (a) Ovarian stroma in the control group showing follicles within normal limits and mild inflammatory cell infiltration (H&E, ×200). (b) Ovarian stroma demonstrating marked congestion, inflammatory cell infiltration, and degenerative follicular structures in the ischemia/reperfusion group (H&E, ×100). (c) Ovarian stroma showing prominent hemorrhage and congestion in the ischemia/reperfusion group (H&E, ×100). (d) Degenerative follicular structures with mild edema and congestion in the enoxaparin group (H&E, ×200). (e) Ovarian stroma showing marked congestion, inflammatory cell infiltration, edema, and degenerative follicular structures in the ischemia/reperfusion group (H&E, ×100).

Source: Elaborated by the authors.

H&E-stained ovarian tissue sections from all study groups were examined and photographed using a light photomicroscope (Olympus BX-51; Olympus Co., Tokyo, Japan) at ×10 and ×20 magnifications. Histopathological evaluation was performed by assessing morphological alterations associated with I/R injury (Fig. 2).

A semi-quantitative scoring system, adapted from previously published studies, was used to grade the severity of histopathological findings^12^. In this scoring system, observed histopathological changes were classified by the severity of I/R injury and scored on a scale of 1–4, as outlined in Table 1.

**Table 1:** Histopathological morphological changes and scoring system

Histopathological and morphological changes	Score			
	1 (< 25%)	2 (≥ 25%)	3 (≥ 50%)	4 (≥ 75%)
Inflammatory cell infiltration	Absent	Mild infiltration	Moderate infiltration	Severe infiltration
Vascular congestion	Absent	Mild	Moderate	Severe
Hemorrhage	Absent	Mild	Moderate	Severe
Edema	Absent	Mild	Moderate	Severe
Degenerative follicles	Absent	Mild	Moderate	Severe

Source: Elaborated by the authors.

### Assessment of oxidative stress and antioxidant status

Serum TAS was measured using a commercially available colorimetric assay kit (Rel Assay Diagnostics, Türkiye) based on the method described by Erel et al.^13^. This method is based on the reduction of the dark blue–green 2,2’-azino-bis (3-ethylbenzothiazoline-6-sulfonic acid) (ABTS) radical to a colorless form in the presence of antioxidants13. For TAS measurement, 100 μL of reagent 1 (R1) was mixed with 10 μL of serum sample, and the initial absorbance (A1) was measured at 660 nm using a microplate reader (BioTek Synergy^TM^ HTX Multi-Mode Reader). Subsequently, 10 μL of reagent 2 (R2) was added, and after 10 minutes of incubation, the second absorbance (A2) was recorded. Ascorbic acid was used as the standard, and TAS values were expressed as mmol Trolox equivalents/L.

Serum TOS was determined photometrically using the method developed by Erel et al.^14^, which is based on the oxidation of ferrous ion–chelator complexes to ferric ions by oxidants present in the sample. The ferric ions subsequently form a colored complex with xylenol orange in an acidic medium. Briefly, 225 μL of reagent 1 (R1) was mixed with 35 μL of the serum sample, and the initial absorbance (A1) was measured at 560 nm. Then, 11 μL of reagent 2 (R2) was added, and after 10 minutes of incubation at room temperature, the second absorbance (A2) was recorded. Hydrogen peroxide (H2O2) was used as the calibrator. TOS values were normalized to protein concentration and expressed as μmol H_2_O_2_ equivalents/L.

The oxidative stress index (OSI) was calculated as the ratio of TOS to TAS and expressed as arbitrary units (AU). All measurements were performed in duplicate, and the mean values were used for statistical analysis.

### Statistical analysis

Statistical analyses were performed using the Statistical Package for the Social Sciences (SPSS) software, version 23.0 (IBM Corp., Armonk, NY, United States of America). Descriptive statistics were used to summarize the data obtained from the study groups. Comparisons among experimental groups were conducted using one-way analysis of variance (ANOVA), followed by Tukey’s honestly significant difference (HSD) post hoc test for multiple comparisons when appropriate. For data that did not meet the assumptions of normality, the non-parametric Kruskal–Wallis’ test was applied. A *p*-value of less than 0.05 was considered statistically significant.

## Results

No animals were lost during the experimental period. Therefore, the final analysis included all 30 rats.

Histopathological evaluation of H&E-stained ovarian tissue sections revealed no statistically significant differences between the I/R + enoxaparin group and the I/R group for any of the assessed parameters (*p* > 0.05). However, except for hemorrhage, all histopathological injury parameters, including inflammatory cell infiltration, vascular congestion, edema, and degenerative follicles, showed numerically lower scores in the I/R + enoxaparin group than in the I/R group.

When the I/R groups were analyzed separately against the control group, all histopathological changes were significantly higher in both I/R groups than in the control group (*p* < 0.05).

The total histopathological injury scores were calculated by summing all evaluated parameters and were 58 for the control group, 123 for the I/R group, and 114 for the I/R + enoxaparin group. The control group exhibited significantly lower total histopathological scores compared with both I/R groups (*p* < 0.05). Although the total injury score was numerically lower in the I/R + enoxaparin group compared with the I/R group, this difference did not reach statistical significance (*p* > 0.05) (Table 2).

**Table 2:** Comparison of histopathological scores of the groups (one-way analysis of variance; differences between groups were analyzed using post-hoc Tukey’s honestly significant difference).

	Group	n	Mean ± SD	Min–Max	Post-hoc p-value
Inflammatory cell infiltration	1	10	1.0 ± 0.0	1–1	1–2: < 0.05* 1–3: > 0.05 2–3: > 0.05
	2	10	1.7 ± 0.6	1–3	
	3	10	1.4 ± 0.6	1–3	
Vascular congestion	1	10	1.7 ± 0.7	1–3	1–2: < 0.05* 1–3: < 0.05* 2–3: > 0.05
	2	10	3.2 ± 0.8	2–4	
	3	10	2.9 ± 0.7	2–4	
Hemorrhage	1	10	1.1 ± 0.3	1–2	1–2: < 0.05* 1–3: < 0.05* 2–3: > 0.05
	2	10	2.6 ± 1.2	1–4	
	3	10	2.9 ± 0.7	2–4	
Edema	1	10	1.0 ± 0.0	1–1	1–2: < 0.05* 1–3: < 0.05* 2–3: > 0.05
	2	10	2.5 ± 0.5	2–3	
	3	10	2.3 ± 0.7	1–3	
Degenerative follicles	1	10	1.0 ± 0.0	1–1	1–2: < 0.05* 1–3: < 0.05* 2–3: > 0.05
	2	10	2.3 ± 0.9	1–4	
	3	10	1.9 ± 0.6	1–3	

* Statistically significant (p < 0.05).

Source: Elaborated by the authors.

TAS and OSI showed statistically significant differences among all pairwise group comparisons (group 1–2, group 1–3, and group 2–3; all p < 0.05). TAS was the lowest in the I/R group and significantly increased in the I/R + enoxaparin group, whereas OSI reached the highest levels in the I/R group and was significantly reduced following enoxaparin administration.

**Figure 3:**
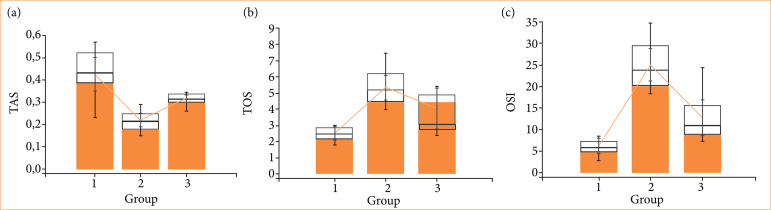
Enoxaparin’s effect on total antioxidant status (TAS), total oxidant status (TOS), and oxidative stress index (OSI). (a) Effect of enoxaparin on TAS levels. (b) Effect of enoxaparin on TOS levels. (c) Effect of enoxaparin on OSI values. One-way analysis of variance–Kruskal–Wallis’ test.

Source: Elaborated by the authors.

TOS was the highest in the I/R group and differed significantly between the control group and both I/R groups (group 1–2 and group 1–3; *p* < 0.05), but no statistically significant difference was observed between the I/R and I/R + enoxaparin groups (*p* > 0.05) (Fig. 3).

## Discussion

This study demonstrated that enoxaparin administration reduced histopathological injury and oxidative stress in ovarian tissue subjected to I/R. Compared with the I/R group, enoxaparin treatment was associated with decreased total histopathological damage scores, increased TAS, and reduced TOS and OSI, but no statistically significant histopathological improvement was observed. These findings are consistent with previous experimental studies demonstrating that agents with anticoagulant and antioxidant properties can attenuate I/R-induced oxidative damage in ovarian tissue. Previous experimental evidence indicates that co-treatment with enoxaparin and α-lipoic acid reduces oxidative stress parameters and histopathological damage in a rat model of ovarian torsion, thereby highlighting a potential role of enoxaparin in regulating oxidative homeostasis and maintaining tissue integrity during I/R injury^15^.

The pathophysiology of I/R injury is known to involve excessive production of ROS upon reperfusion, which overwhelms endogenous antioxidant defenses and leads to cellular damage through lipid peroxidation and inflammatory processes^16-18^. Beyond its well-established anticoagulant activity, enoxaparin has been reported to exert pleiotropic effects that may contribute to tissue protection during I/R injury. Experimental evidence indicates that enoxaparin attenuates microvascular dysfunction, limits endothelial injury, and reduces inflammatory cell infiltration following reperfusion, thereby mitigating secondary tissue damage. These mechanisms are particularly relevant in ovarian ischemia, in which microcirculatory impairment and inflammatory responses play a pivotal role in follicular injury and depletion of ovarian reserve^18-20^. In this context, the increase in TAS and the concomitant reduction in TOS and OSI following enoxaparin treatment suggest modulation of redox balance after I/R injury. These findings are consistent with the well-established role of oxidative stress in ovarian I/R injury, in which excessive ROS production disrupts redox balance, as reflected by alterations in TAS, TOS, and OSI levels, ultimately contributing to cellular damage^21,22^. Together with the histopathological findings, these results indicate that the protective effects of enoxaparin may be partially mediated by attenuation of oxidative stress and preservation of tissue integrity^15^.

Protective effects mediated through attenuation of oxidative stress have been reported with antioxidant agents in ovarian I/R models, further supporting the pivotal role of redox imbalance in ovarian I/R–induced tissue injury^23^. In this regard, Granger and Kvietys^18^ comprehensively described I/R injury as a process largely driven by excessive ROS generation during reperfusion, leading to microvascular dysfunction, endothelial activation, increased vascular permeability, and inflammatory cell recruitment, which together exacerbate tissue damage.

One limitation of the present study is that the 1-hour ischemia followed by 1-hour reperfusion model may not fully reflect the complexity and heterogeneity of clinical ovarian I/R scenarios. In addition, only a single enoxaparin dosing protocol was evaluated, and the pharmacokinetics and optimal dosing regimen were not specifically investigated. Therefore, the relationship between enoxaparin dose, tissue exposure, and protective efficacy could not be fully elucidated. Another important limitation is the absence of long-term functional ovarian reserve markers, such as anti-Müllerian hormone levels or follicular counts, which may better reflect the clinical relevance of ovarian protection. Furthermore, the relatively short duration of I/R may have limited the ability to detect more pronounced histopathological differences between groups. Future studies employing different dosing strategies, extended I/R durations, and long-term functional assessments are warranted to better characterize the role of enoxaparin in ovarian I/R injury.

## Conclusion

The present findings demonstrate that enoxaparin administration attenuates I/R–induced ovarian injury by reducing histopathological damage and improving oxidative stress parameters. These results suggest that enoxaparin may exert a protective effect on ovarian tissue, potentially through modulation of oxidative balance, although further studies are required to clarify its underlying mechanisms and clinical relevance.

## Data Availability

The datasets used and/or analyzed during the current study are available from the corresponding author on reasonable request.
